# A Pilot Study of the Feasibility of In-Ear EEG for Long-Term Temporal Lobe Brain Wave Monitoring

**DOI:** 10.3390/s26196343

**Published:** 2026-10-08

**Authors:** Andy Ho Wing Chan, Ryan C. Emadi, Andy Lin, Mark J. Schroeder, Ravinderjit Singh, Riaz Shaik, Mary-Catherine George, Jack Gomberg, Onome Eka, Toni Kavanagh, Ji Yeoun (Jenna) Yoo, Joanna C. Jen, Ali Tajer, Anuradha Singh, Mijail Serruya, Achintya K. Bhowmik, Muhammad A. Parvaz

**Affiliations:** 1 Department of Neurology, Rockefeller Neuroscience Institute, West Virginia University, Morgantown, WV 26506, USA; 2 Department of Psychiatry, Icahn School of Medicine at Mount Sinai, New York, NY 10029, USAmuhammad.parvaz@mssm.edu (M.A.P.); 3 School of Medicine, West Virginia University, Morgantown, WV 26506, USA; rce00003@mix.wvu.edu; 4 Starkey Hearing Technologies, Eden Prairie, MN 55344, USAmark_schroeder@starkey.com (M.J.S.);; 5 Department of Neurology, Icahn School of Medicine at Mount Sinai, New York, NY 10029, USA; mary-catherine.george@mssm.edu (M.-C.G.); onome.eka@mssm.edu (O.E.);; 6 Department of Electrical, Computer, and Systems Engineering, Rensselaer Polytechnic Institute, Troy, NY 12180, USA; 7 Department of Neurology, Yale School of Medicine, New Haven, CT 06510, USA; 8 Department of Neurosurgery, Yale School of Medicine, New Haven, CT 06510, USA; 9 Raphael Center for Neurorestoration, Vickie and Jack Farber Institute for Neuroscience, Jefferson Health, Philadelphia, PA 19107, USA; mijail.serruya@jefferson.edu; 10 Department of Neuroscience, Icahn School of Medicine at Mount Sinai, New York, NY 10029, USA; 11 Center for Artificial Intelligence and Human Health, Icahn School of Medicine at Mount Sinai, New York, NY 10029, USA

**Keywords:** in-ear EEG, epilepsy, patient comfort, remote patient monitoring, digital health monitoring, wearable EEG technology

## Abstract

**Background:** Traditional scalp electroencephalography (EEG), though clinically validated, presents significant limitations, including lengthy setup, patient discomfort, and limited mobility. These factors hinder its utility for long-term ambulatory monitoring. In-ear EEG offers a promising alternative by leveraging the ear canal’s anatomical proximity to the temporal lobes and natural mechanical stability. To address this, we developed SeizEAR, a standardized, three-size in-ear EEG system using paste-based electrodes with cross-ear referencing. **Methods:** This pilot study evaluated SeizEAR devices featuring small, medium, and large earpieces with superior and inferior paste-based electrodes in each ear, configured in a cross-ear reference scheme. It included five healthy adults who underwent recording sessions of approximately three hours (2.4 to 3.3 h). Electrode impedance was monitored against the acquisition system’s threshold indicator, with simultaneous scalp EEG from T7/T8 electrodes serving as the reference. Participants rated comfort on a 0–5 Likert scale every 30 min. **Results:** In-ear recordings showed moderate, band-dependent correspondence with ipsilateral temporal scalp electrodes, strongest in the delta and alpha bands and weakest in beta, and correspondence was consistently higher against a geometrically matched T7–T8 bipolar derivation than against a single referenced electrode. In-ear alpha reactivity to eye closure was present but attenuated relative to scalp. Comfort ratings remained high over the final two hours of wear, with no reported adverse events. **Conclusions:** SeizEAR acquired in-ear EEG that corresponded moderately with temporal scalp EEG and was well-tolerated over multi-hour wear in healthy adults. These preliminary findings support further development of a standardized in-ear EEG system and require confirmation in an adequately powered sample.

## 1. Introduction

Electroencephalography (EEG) remains an essential neurophysiological modality for the assessment of functional brain activity across a wide spectrum of neurological and psychiatric disorders. It provides direct measurement of cortical oscillations with millisecond temporal resolution, thereby offering critical insights into both normal and pathological brain dynamics. Conventional scalp EEG, while clinically proven, presents significant practical challenges that limit patient acceptability and real-world monitoring capabilities. Conventional EEG setup is time-consuming, often requiring up to an hour for electrode placement with meticulous skin preparation and conductive paste application. The extensive electrode array, typically 19 or more in the standard 10–20 system, restricts patient mobility, creates social stigma due to visibility, and frequently results in data degradation from movement artifacts during extended recordings. A survey of neurologists highlighted strong medical interest in wearable EEG technology to address these limitations, though development faces key challenges, particularly in electrode technology where current systems need significant improvement to ensure reliable, long-term contact in a discreet, user-friendly manner [1].

The internal ear canal represents a promising EEG acquisition site, offering distinct anatomical advantages over conventional scalp-based methods. Its proximity to the temporal lobes, critical brain regions housing key neural networks, enables potentially enhanced signal capture from these areas [2,3,4,5,6,7,8,9,10]. The ear canal provides a naturally stable, shielded environment that minimizes movement artifacts and hair interference while offering discreet, comfortable electrode positioning, especially when compared to an around-the-ear approach [11,12,13,14]. While numerous attempts have been made to develop around-the-in-ear EEG designs, the internal ear canal approach offers superior signal stability, reduced artifact susceptibility, and enhanced discreteness by eliminating issues such as hair interference and electrode displacement that commonly affect external ear-mounted systems [6,11,15]. Recent research has established the fundamental viability of in-ear EEG, demonstrating that ear canal recordings can reflect cortical activity with reasonable fidelity [5,7,12,16,17]. Subsequent quantitative studies have shown auditory event-related potentials were visible in the in-ear EEG signal with moderate-to-high effect sizes, though amplitudes were reduced by 21–44% relative to scalp recordings [18]. Others demonstrated that in-ear EEG demonstrated substantial agreement with scalp EEG during rest and nap states [5].

Building on this foundation, clinical validation has expanded across diverse neurological and psychiatric applications, demonstrating in-ear EEG’s broad utility beyond its traditional epilepsy focus. In epilepsy monitoring, in-ear EEG has been found to detect focal temporal seizures with visual review yielding the same seizures as scalp EEG and no loss of sensitivity [3,19]. Others extended this to more refractory cases, showing in-ear EEG captured approximately 87.5% of seizures seen on intracranial EEG, often identifying epileptic events exceeding patients’ self-reports [19]. For sleep disorders, in-ear EEG has been shown with polysomnography, achieving Cohen’s kappa agreement of ~0.65–0.71 with scalp recordings in sleep staging tasks, with sensitivity/specificity for deep sleep detection reaching ~88%/78% [5]. This capability opens possibilities for monitoring conditions like sleep apnea, insomnia, and circadian rhythm disorders [20,21]. Cognitive and psychiatric applications have also shown promise, with even around-in-ear EEG demonstrating the ability to track mental workload during working memory tasks, achieving ~95% accuracy in classifying high versus low cognitive load with 12-channel configurations [12]. In affective monitoring, single-channel in-ear EEG achieved ~71% accuracy on basic emotion and ~73% on arousal classification, suggesting potential applications in depression monitoring, anxiety assessment, and ADHD evaluation [4].

However, a substantial portion of the literature focuses on custom-made in-ear EEG devices tailored to individual patients through 3D scanning and molding processes [2,6,19]. While these custom approaches demonstrate excellent signal quality and fit, they present significant barriers to widespread clinical adoption, requiring specialized equipment, time-intensive fabrication processes, and individual ear modeling that limit scalability and increase costs. A more practical approach involves developing standardized, off-the-shelf in-ear EEG devices available in multiple sizes that eliminate the custom fabrication process while maintaining comfort and signal quality. This shift would enable immediate deployment for remote brain monitoring without the delays and costs associated with individual ear modeling. Studies support the feasibility of this approach: subjects generally report high comfort and tolerance for multi-hour recordings with generic in-ear EEG systems that do not have to be custom-fitted to the mold of each individual patient’s ear [20], with an epilepsy-oriented study showing patients wore in-ear EEG systems for a median of 13 h continuously with high tolerance levels [19]. Importantly, sleep monitoring studies found no significant difference in performance between custom versus generic earpieces, suggesting efficacious functionality across standardized designs [20]. The natural resemblance to common audio earbuds makes in-ear EEG socially unobtrusive and suitable for everyday activities, combining ergonomic convenience with clinical utility.

The translation of in-ear EEG from research prototype to scalable clinical tool requires moving beyond custom-fabricated earpieces toward standardized, off-the-shelf designs. Prior work has demonstrated the feasibility of generic in-ear EEG in several important forms. Kidmose and colleagues established the foundational feasibility of generic earpiece-based in-ear EEG as early as 2013 [22]. Goverdovsky et al. subsequently introduced viscoelastic memory foam substrates with conductive cloth electrodes, demonstrating that a one-size-fits-most design could achieve stable electrode–skin contact through passive conformance to the ear canal without requiring conductive gel or custom molding [23]. Kaveh et al. further advanced this space with a wireless, multi-electrode, user-generic platform using dry electrodes fabricated via vacuum forming and spray coating, demonstrating alpha modulation comparable to scalp EEG [24]. These contributions established that generic in-ear EEG is technically viable and that off-the-shelf deployment need not compromise signal quality.

SeizEAR builds on this foundation while introducing a distinct design approach that addresses limitations not fully resolved by prior generic systems. Rather than relying on dry or saline-wetted cloth electrodes, SeizEAR employs paste-based Ag-AgCl electrodes within a standardized three-size framework. This combination is deliberate: paste-based contacts are intended to provide a low-impedance interface suitable for direct comparison against clinical-grade scalp EEG recordings, without requiring individual ear molding. SeizEAR further implements a cross-ear referencing scheme using one superior and one inferior electrode per ear, eliminating dependence on scalp reference electrodes while preserving a simplified two-channel acquisition architecture. Our study evaluates the tolerability, comfort, and preliminary signal characteristics of this design in healthy adults, using concurrent 21-electrode scalp EEG acquisition on a clinical-grade Natus XLTEK platform as the reference standard.

## 2. Method

### 2.1. Aim, Design, and Setting of the Study

This pilot study characterizes the signal correspondence and tolerability of SeizEAR, an in-ear EEG device intended for temporal lobe monitoring, in five healthy adults with concurrent scalp EEG. We hypothesized that in-ear EEG acquired with a cross-ear derivation would show band-dependent correspondence with ipsilateral temporal scalp EEG, reproduce the direction of the eyes-closed alpha response, and be tolerated over multi-hour wear. With five participants this hypothesis is evaluated at the participant level, by reporting each participant’s values and the number of participants showing the predicted direction for each comparison; the sample does not support formal statistical inference. The study employs a prospective, comparative and descriptive design with concurrent validation against standard scalp EEG electrodes, conducted in a controlled laboratory setting.

### 2.2. Participants

Five healthy adult volunteers (eligible age range 18–70 years; enrolled participants aged 21 to 29 years, two men and three women) were recruited for device validation. All participants provided written informed consent prior to enrollment. Exclusion criteria: Documented history of epilepsy or seizure disorders, known contact dermatitis or skin hypersensitivities, active otitis externa or media, sensorineural or conductive hearing loss, cognitive impairment precluding informed consent, concurrent use of medications affecting neurological function, or any significant comorbidities. The study was conducted in accordance with the Declaration of Helsinki, and approved by the Institutional Review Board Program for the Protection of Human Subjects of Icahn School of Medicine at Mount Sinai (protocol code 24-00433 and date of approval 25 March 2025). All participants provided written informed consent.

### 2.3. Device Description

The SeizEAR prototype consists of silver-chloride (Ag-AgCl) electrodes fabricated using conductive ink (Creative Materials, Ayer, MA, USA) applied to insulated copper wire, secured within channels embedded in a 3D-printed polymer substrate and encapsulated in medical-grade earbud foam (Smooth-On, Macungie, PA, USA). Color-coded electrode buds are secured via adjustable silicone retainers. A custom 3D-printed handle, made of black SLA resin (Allied Photochemical, Macomb, MI, USA), was fabricated using the PRO 4K UV printer (Asiga, NSW, Australia). This handle serves as the primary chassis, providing insertion depth control, structural organization of electrode leads, and an acoustic vent via a central hollow core connected to flexible tubing, which reduces occlusion effects and preserves ambient sound transmission. A flexible retaining mold fitted over the handle enhances positional stability and accommodates varying ear canal geometries through an adjustable sliding and rotational interface. Three standardized sizes (small, medium, large) are available to accommodate most ear canal anatomies without custom fabrication. Each earpiece consists of a 17 mm handle carrying a foam bud with a superior and an inferior electrode. The small bud measures 12.7 mm in length, 7.6 mm in height and 5.6 mm in width, and the medium bud 12.7 by 10.7 by 7.6 mm; total length from handle base to bud tip is approximately 30 mm for both. Each electrode presents a contact surface of 9.7 by 3.0 mm, approximately 29 mm^2^. Insertion depth is set by the bud, so the electrodes sit within the outer 13 mm of the canal. Earpiece size was selected by fit: a bud was inserted and accepted if it seated in the canal without falling out and the acquisition display showed an adequate signal, and a different size was tried otherwise. The same size was used on both ears, small in two participants and medium in three (Table 1). A fresh earpiece was used for each participant. Canal cleansing, paste application and insertion took approximately two to three minutes per participant, with paste applied to the electrode surfaces.

SeizEAR electrodes were connected via hardware leads to a Natus XLTEK 32-channel amplifier, operating at a sampling rate of 1024 Hz, and recorded through the Natus XLTEK Brain Monitor EEG and Sleep System. This configuration leverages an established clinical recording platform, obviating the need for a standalone battery or wireless transmission system in this pilot study. Electrode impedance was monitored continuously on the Natus system’s threshold indicator, which reports whether each electrode is within the system’s acceptance range (below 50 kΩ) but does not display or export numerical impedance values. All SeizEAR electrodes remained within range throughout every session. Numerical impedance values and impedance time courses are therefore not available from this study. **Recording Procedure:** Prior to electrode placement, the external auditory canal was examined by a physician together with the study EEG technician, and the ear canal was cleansed with isopropyl alcohol. Conductive paste (Ten20, Weaver and Company, Aurora, CO, USA) was applied to the silver-chloride electrodes before gentle insertion and securing within the ear canal. Paste was applied once at placement and was not reapplied during any session. Concurrent scalp EEG was recorded using a standard 21-electrode 10–20 system montage: C3, C4, Cz, F3, F4, F7, F8, Fz, Fp1, Fp2, Fpz, O1, O2, Oz, P3, P4, P7, P8, Pz, T7, T8. This was also connected to the Natus XLTEK amplifier.

The protocol permitted recording sessions of up to six hours. All five sessions were concluded by the study team between 2.4 and 3.3 h (Table 1); no session was terminated for signal loss, impedance failure or participant discomfort.

### 2.4. Recording Protocol

The experimental session consisted of three consecutive phases: calibration, resting-state EEG task, and video-viewing task. Calibration, comprising electrode placement, impedance verification and signal settling, took approximately 2000 s.

During the resting-state EEG task, participants sat comfortably in a quiet, dimly lit room and were instructed to relax and stay awake. EEG was recorded continuously for 10 min, split into two 5 min segments: eyes open (EO), fixated on a central cross, and eyes closed (EC), during which they remained relaxed and alert. This paradigm was designed to elicit the well-established alpha rhythm modulation, where alpha power (8–13 Hz) typically increases during eyes-closed conditions compared to eyes-open conditions, providing a standard neurophysiological validation of EEG signal quality [2,15].

Following this, participants passively viewed video content for the remainder of the session.

Sessions lasted between 2.4 and 3.3 h. Concurrent standard scalp EEG including recordings at T7 and T8 sites were used as reference standards for signal comparison. Both scalp EEG and in-ear EEG electrodes were connected to the Natus XLTEK Brain Monitor EEG & Sleep System to ensure synchronized recording. Participant comfort was assessed at baseline, every 30 min during recording, and at session completion using a 0–5 Likert scale, evaluating headset feel, security, tightness, weight, overall comfort, and overall fit. Adverse events were ascertained by participant report, with participants instructed to report discomfort immediately, and by direct observation at each scheduled comfort assessment. Comfort scores were acquired every 30 min. Because session length varied between 2.4 and 3.3 h, ratings were aligned to session end and comfort is reported over the final two hours of each session, the window common to all five participants.

### 2.5. Signal Processing and Analysis

EEG data were processed in part using the open-source library MNE [25]. Signals were bandpass filtered between 1 and 40 Hz, and a notch filter was applied at 60 Hz to suppress line noise. Scalp electrodes were re-referenced to the average of all scalp electrodes. For in-ear EEG, the left channel was defined as the left upper electrode referenced to the right lower electrode, and the right channel as the right upper electrode referenced to the left lower electrode. A third scalp derivation, T7 referenced to T8, was computed as a wide bipolar derivation geometrically matched to the cross-ear channel, and every in-ear to scalp comparison is reported against both the average-referenced ipsilateral temporal electrode and this matched derivation.

In addition to this primary “crossed” reference configuration, we analyzed a secondary “ipsilateral” reference configuration, wherein the left upper electrode was referenced to the left lower electrode and the right upper electrode was referenced to the right lower electrode.

Blink artifacts were removed from the scalp EEG using Independent Component Analysis (ICA), implemented in MNE with the “fastica” algorithm [25]. ICA components were visually inspected, and the component exhibiting blink-related morphology was excluded. The EEG signal was then reconstructed with the remaining components. ICA was applied to the scalp EEG only. Scalp EEG is the reference standard in this comparison and therefore received the cleanest processing available, whereas the in-ear signal is the signal under evaluation and was compared as recorded; ICA is in any case not applicable to a two-channel derivation. Any ocular contribution removed from the scalp side alone can lower the reported in-ear to scalp correspondence but not raise it, so the reported values are conservative. Beyond the amplitude-based rejection described below, no artifact-specific correction was applied to the in-ear signal.

Spectral power in the canonical frequency bands delta (1–4 Hz), theta (4–8 Hz), alpha (8–13 Hz), and beta (13–30 Hz) was computed using the multitaper method with four discrete prolate spheroidal sequence (DPSS) tapers, as implemented in MNE [25]. To facilitate comparison between in-ear EEG and scalp EEG, we calculated the proportion of power in each band relative to the total power across all four bands.

For the alpha attenuation analysis, spectral power in the alpha band was computed separately for the eyes-open and eyes-closed conditions, as implemented in MNE23 within our custom-made Python processing pipeline. Alpha attenuation was quantified as the ratio of alpha power during eyes-closed to eyes-open conditions, with higher ratios indicating stronger alpha modulation. This analysis was performed for both in-ear channels; the temporal scalp electrodes T7 and T8; the T7–T8 bipolar derivation; and the occipital and parietal electrodes O1, O2, P7 and P8, to compare alpha reactivity across sites.

Additional signal comparison analyses were performed under the three task conditions EO, EC, and “ALL” which comprise the entire duration of the trial excluding the first 2000 s (calibration time). For each condition, power spectral density (PSD) plots were generated, using the aforementioned MNE taper method from 1 to 40 Hz, and smoothed with a 1-dimensional Gaussian filter (σ = 2) provided by the SciPy Python package (SciPy 1.17.1 and Python 3.11.15).

For each condition, time-series correlations were computed using 5 s sliding windows, each spaced 1 s apart. Before applying sliding windows, individual samples exceeding ±100 µV in either in-ear channel were removed and the remaining samples concatenated. This sample-level rule differs from the window-level rule used for the band-power and coherence analyses described below, and it introduces discontinuities at each excision. Across each 5 s window, the Pearson correlation coefficient **r** and Spearman correlation coefficient **ρ** were computed between the left ear (henceforth, “L-ear”) and T7 scalp electrode recordings, and the right ear (henceforth, “R-ear”) and T8 scalp electrode recordings, and between each ear and the T7–T8 bipolar derivation. Additionally, in order to subjectively evaluate ear–scalp electrode congruence, **r**-coefficients were also computed between L-ear vs. T8, R-ear vs. T7, both L- and R-ear vs. the average between T7 and T8, and both L- and R-ear vs. the average scalp electrode recording.

For each condition, using the same 5 s sliding window, further band-power analysis was computed on the aforementioned delta, theta, alpha, and beta bands. However, this time after applying sliding windows, all windows with any points exceeding ±100 µV were removed. This fixed-amplitude criterion is the standard artifact rejection rule for awake scalp EEG and is implemented in the same form in EEGLAB and ERPLAB [26,27]; amplitudes above this level in a healthy adult at rest usually correspond to muscle activity rather than neuronal activity. Within each window, PSDs were computed on the L-ear, R-ear, T7 and T8 electrodes and on the T7–T8 bipolar derivation, using Welch’s method (SciPy). Band powers for each of the four frequency bands were computed by integrating the PSD across the frequency band in question. The total band power was also computed by integrating across the entire 1–40 Hz spectrum. Band powers were log10-transformed before all correlation and signal-to-noise computations. The band-power correlations between each individual frequency band, as well as the total band power, between L-ear vs. T7, R-ear vs. T8, and each ear vs. T7–T8, were computed with r- and ρ-coefficients. Additionally, coherence values were computed for the same pairs. These were computed within each individual band with the SciPy package, using the formula.

Values are reported across 5 s windows rather than as a single value per session because EEG over a multi-hour recording is not stationary; posture and electrode contact change over time, and 5 s is short enough that the power spectrum is unlikely to change substantially within a window. The standard deviation across windows therefore describes within-session variability. Because windows within a participant are not independent, it is not a measure of between-participant precision and is reported descriptively. Each participant’s own values are shown in Table 2, and for each comparison the results state how many of the five participants showed the predicted direction. Formal significance tests are not reported: with five participants no paired test can yield a two-sided *p* below 0.0625, and a confidence interval from five values would suggest a precision the study does not have.

$$C_{ear,scalp}\left(f\right)=\frac{{\left|{CSD}_{ear,scalp}\left(f\right)\right|}^{2}}{{PSD}_{ear}\left(f\right)\cdot {PSD}_{scalp}\left(f\right)}$$

$$r^{2}=\frac{{\sigma_{corr}}^{2}}{{\sigma_{total}}^{2}}=\frac{{\sigma_{signal}}^{2}}{{\sigma_{signal}}^{2}+{\sigma_{noise}}^{2}}=\frac{P_{signal}}{P_{signal}+P_{noise}}$$

Thus, by using algebraic manipulation, SNR can be defined in terms of **r**^2^ and converted to a logarithmic unit scale, according to the following formula:

$$SNR=10{log}_{10}\left(\frac{P_{signal}}{P_{noise}}\right)=10{log}_{10}\left(\frac{r^{2}}{1-r^{2}}\right)$$

Signal-to-noise ratios were computed for all four frequency bands. This derivation treats scalp EEG as the reference standard and therefore as a noiseless quantity. Because scalp EEG carries its own noise, residual variance attributed to the in-ear signal includes a component properly attributable to the reference, and the reported SNR values are conservative lower bounds. Because SNR depends on r^2^, it does not distinguish positive from negative correlation, and signed r is reported alongside it.

## 3. Results

### 3.1. Device Construction and Design Features

**Figure 1:**
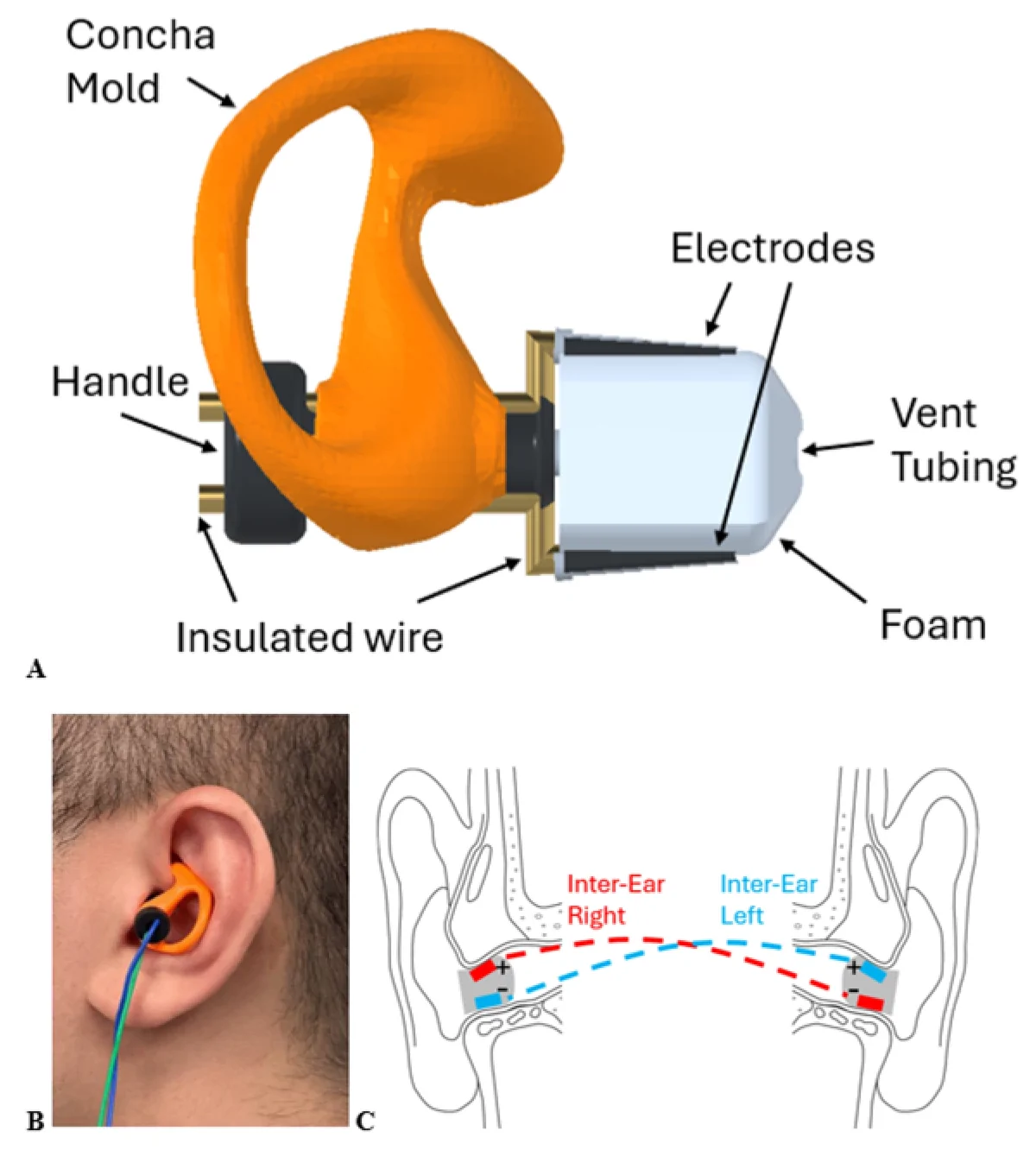
The SeizEAR device. (**A**): A three-dimensional diagram of the in-ear EEG design. (**B**): A SeizEAR device placed in an ear. (**C**): The conceptual design of cross-ear referencing.

The SeizEAR device consisted of multiple integrated components designed to optimize signal acquisition and user comfort (Figure 1A). The recording electrodes were fabricated using silver–silver-chloride (Ag-AgCl) conductive ink (Creative Materials, Ayer, MA, USA) applied to insulated copper wire. Each wire was secured within a channel embedded in a 3D-printed polymer substrate, which was subsequently encapsulated within medical-grade earbud foam (Smooth-On, Macungie, PA, USA) during the molding process.

A custom 3D-printed handle served as the primary chassis and fulfilled several functional roles. Its length and rigidity enabled proper insertion depth and allowed for easy removal from the ear canal. Two parallel wire channels within the handle stabilized the electrode leads and maintained structural organization. A central hollow core in the handle was connected to flexible tubing that extended through the foam insert. This tubing provided mechanical support while allowing flexibility to accommodate varying ear canal anatomies. Additionally, the hollow channel created an acoustic vent that reduced occlusion effects and allowed ambient sound to pass through the device, contributing to a more natural auditory experience (Figure 1B).

To improve positional stability, a flexible retaining mold was fitted over the handle. This mold enhanced device retention within the ear and helped secure the electrode leads, while its adjustable sliding and rotational interface enabled accommodation of diverse ear geometries.

### 3.2. EEG Signal

**Figure 2:**
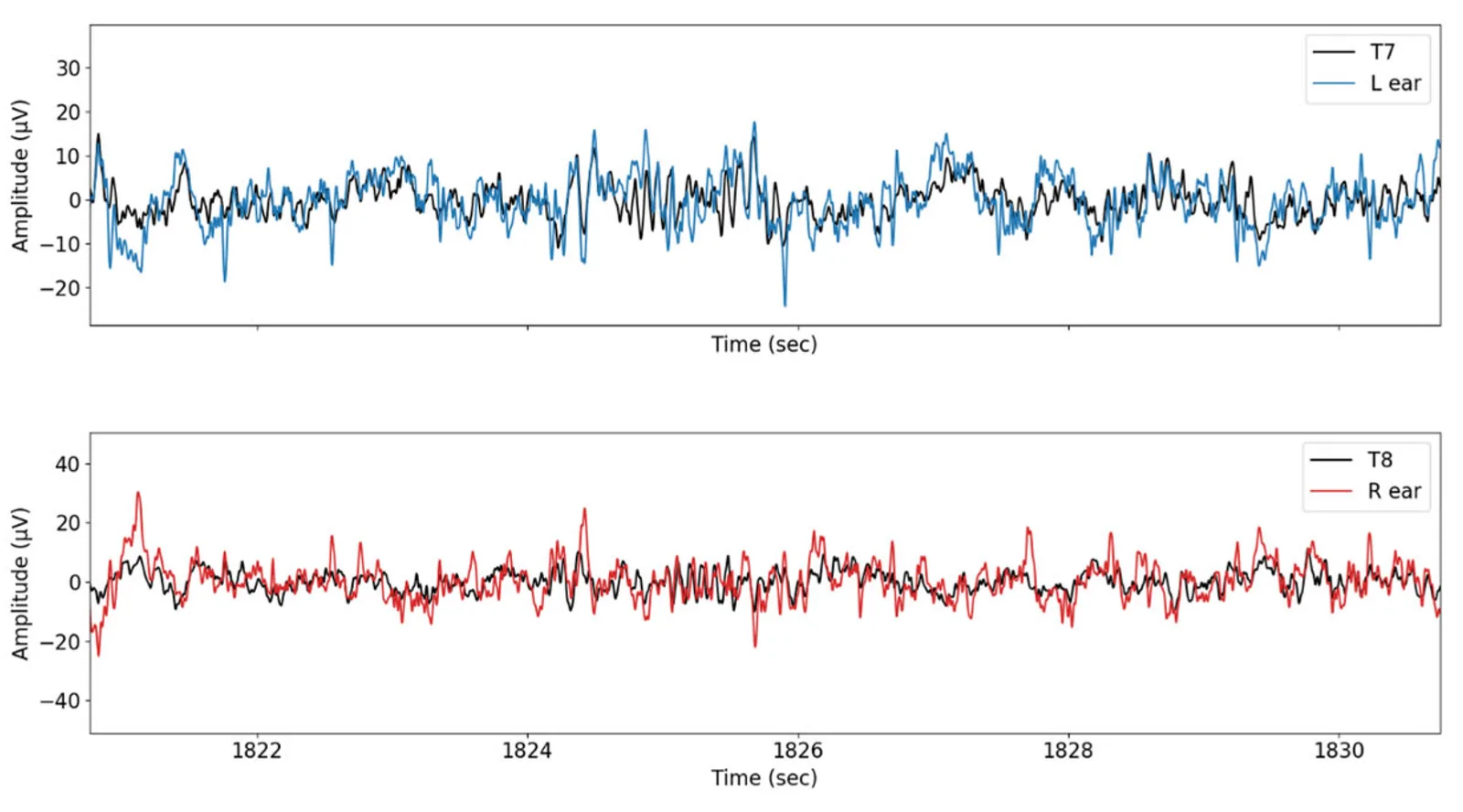
Top panel: A sample time frame demonstrating the blue (left ear) SeizEAR electrode superimposed onto the T7 scalp electrode signal. Bottom panel: The red (right ear) SeizEAR electrode superimposed onto the T8 scalp electrode signal.

As shown in Figure 2, the recorded waveforms exhibited characteristic EEG patterns consistent with cortical activity from the temporal lobes. Each in-ear electrode was referenced to the contralateral lower-ear electrode in a cross-ear montage, as depicted in Figure 1C.

**Figure 3:**
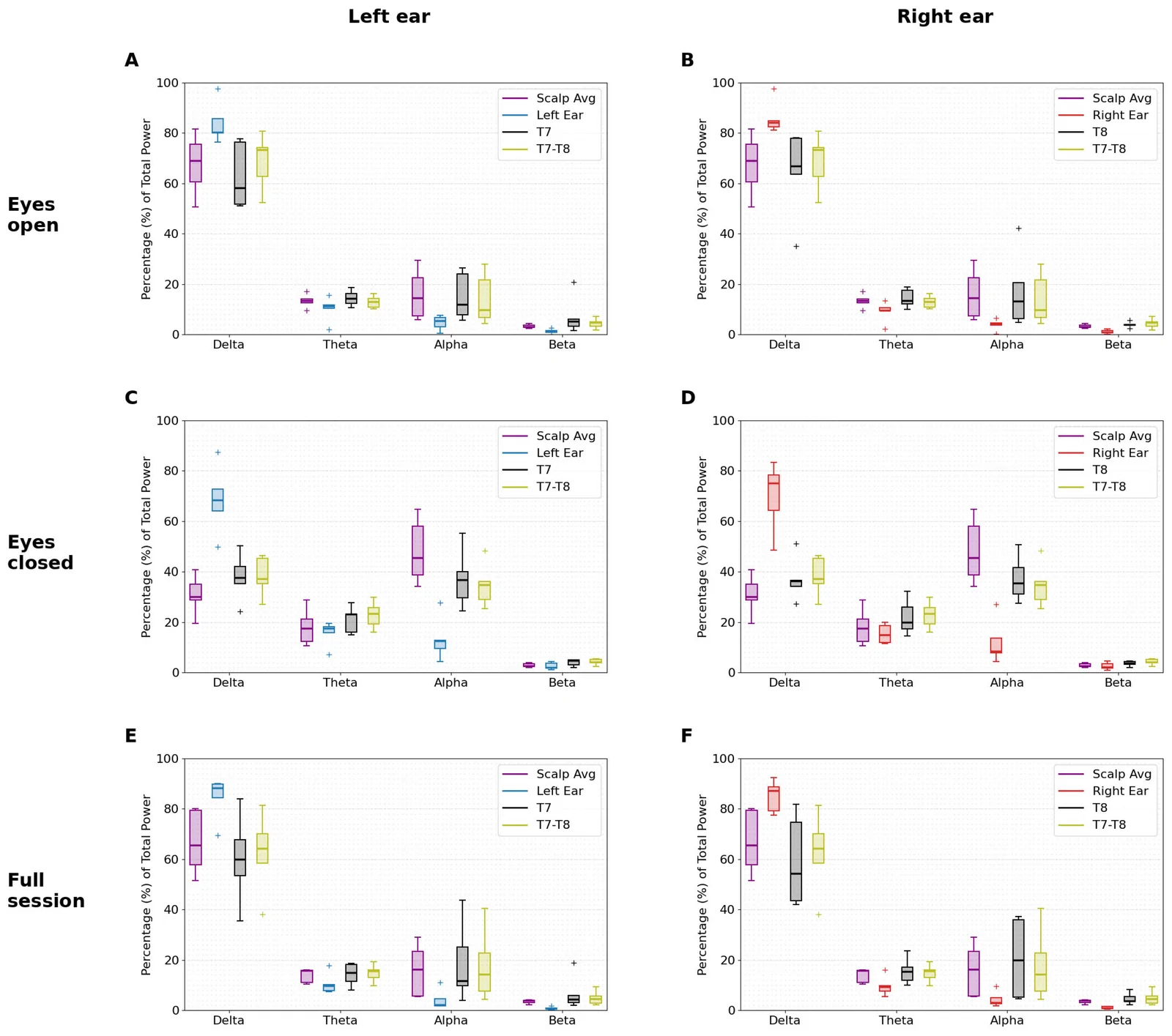
Relative band power in the delta, theta, alpha and beta bands as a percentage of total 1–40 Hz power, across five participants, during eyes open (**A**,**B**), eyes closed (**C**,**D**) and the full session (**E**,**F**). Left column: left in-ear channel with T7; right column: right in-ear channel with T8. Each panel also shows the T7–T8 bipolar derivation, geometrically matched to the cross-ear channel, and the scalp average. The boxes span the interquartile range across participants, the line is the median, the whiskers extend to the most extreme values within 1.5 times the interquartile range, and the crosses are values beyond that.

Figure 3 shows the relative band-power distribution across canonical frequency bands (delta: 1–4 Hz; theta: 4–8 Hz; alpha: 8–13 Hz; beta: 13–30 Hz) for each in-ear channel, the ipsilateral temporal electrode, the T7–T8 bipolar derivation and the scalp average, in the eyes-open, eyes-closed and full-session conditions, with one box per derivation summarizing the five participants. Band power was expressed as a percentage of the total power within the 1–40 Hz range. Compared with every scalp derivation, the in-ear channels carried a substantially higher proportion of delta power (median approximately 87% across the full session against approximately 60% at T7 and 64% at T7–T8) and correspondingly less alpha and beta. The matched T7–T8 derivation did not close this gap: its spectral distribution resembled T7 and the scalp average rather than the in-ear channels, so the difference in spectral composition is a property of the in-ear electrode rather than of the derivation geometry. Theta power was comparable across all derivations. With eye closure the alpha proportion rose at every derivation, from approximately 5% to 11% in the in-ear channels and from approximately 15% to 45% in the scalp average.

### 3.3. Alpha Attenuation Task

During the eyes-open and eyes-closed paradigms, SeizEAR demonstrated detectable alpha modulation patterns in the same direction as scalp EEG recordings. Figure 3C,D show the alpha proportion of total power rising with eye closure at every derivation, including both in-ear channels. Table 2 reports the eyes-closed to eyes-open alpha-power ratio for each participant at both in-ear channels; the temporal scalp electrodes T7 and T8; the T7–T8 bipolar derivation; and the occipital and parietal electrodes O1, O2, P7 and P8. Median ratios were 1.15 for the left ear and 1.49 for the right ear, against 2.54 at T7; 2.37 at T8; 1.88 for the T7–T8 bipolar derivation; and 6.51, 5.45, 4.99 and 4.81 at O1, O2, P7 and P8. A ratio above 1 was observed at the left in-ear channel in three of five participants and at the right in four of five. In every participant who produced an alpha response, occipital and parietal reactivity exceeded temporal reactivity, and in-ear reactivity was lower than either and closest to the T7–T8 bipolar derivation. Participant 1 showed no eyes-closed alpha enhancement at any electrode, including O1 and O2; Participant 3 showed a normal scalp response with in-ear ratios below 1 in both ears.

### 3.4. Spectral Comparison

**Figure 4:**
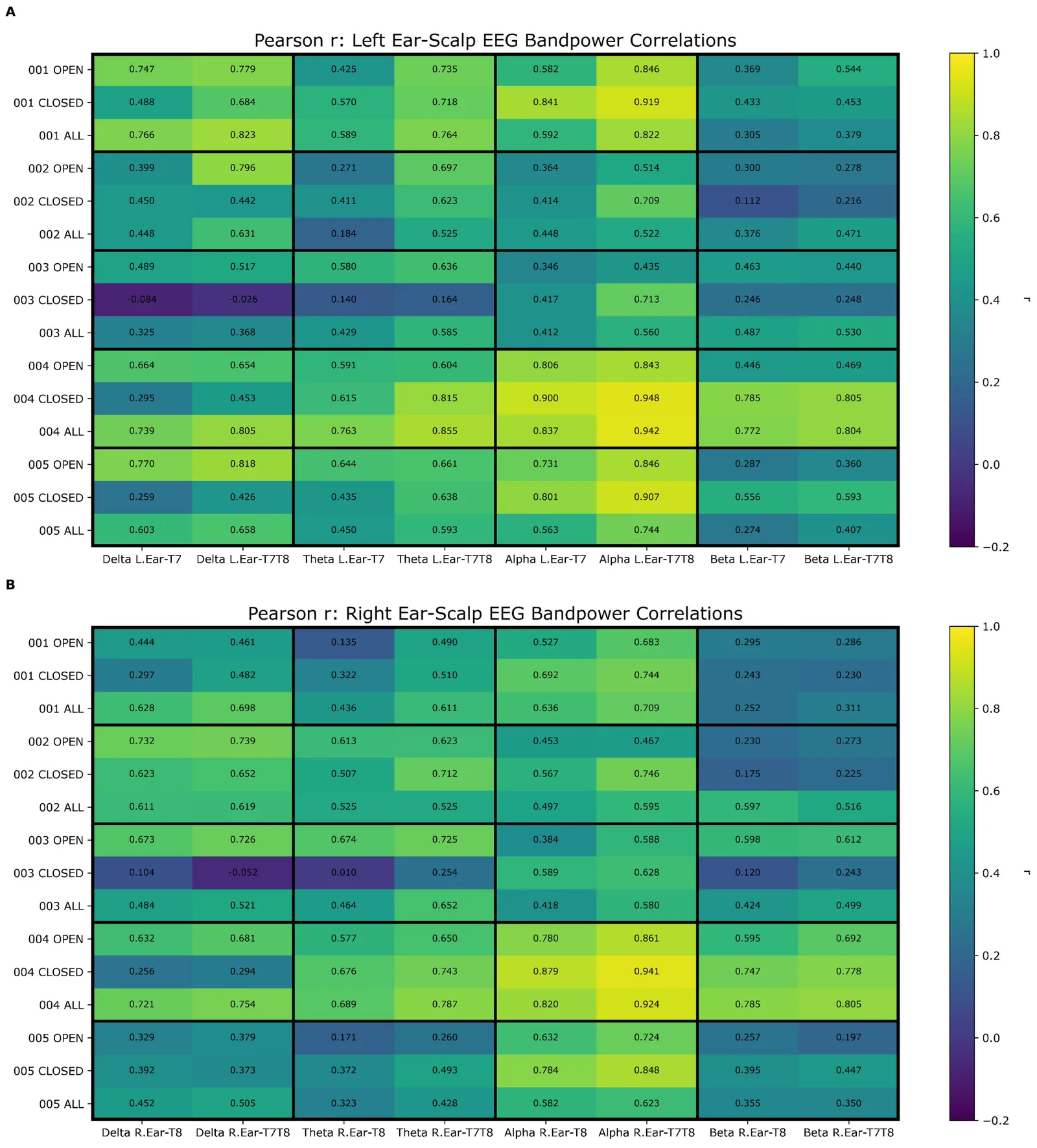
The signed Pearson correlation of log band power between each in-ear channel and scalp EEG, for every participant (001 to 005) and condition (EO, EC, ALL) in the delta, theta, alpha and beta bands. (**A**) Left ear. (**B**) Right ear. Within each band, the first column is the in-ear channel against the common-average-referenced ipsilateral temporal electrode (T7 or T8) and the second column is the same in-ear channel against the T7–T8 bipolar derivation, which is geometrically matched to the cross-ear channel.

**Figure 5:**
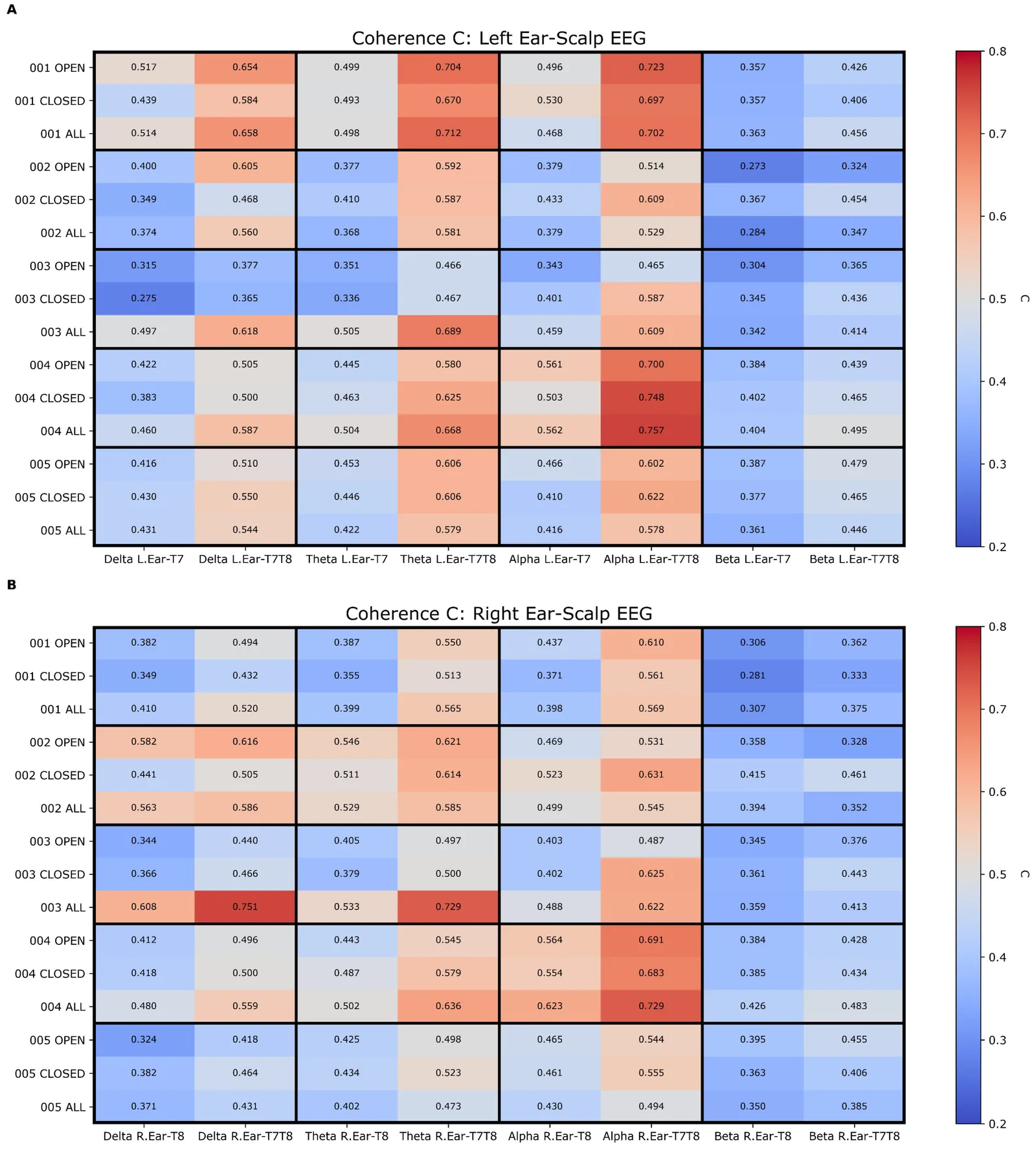
The magnitude-squared coherence between each in-ear channel and scalp EEG, for every participant (001 to 005) and condition (EO, EC, ALL) in the delta, theta, alpha and beta bands. (**A**) Left ear. (**B**) Right ear. Within each band, the first column is the in-ear channel against the common-average-referenced ipsilateral temporal electrode (T7 or T8) and the second column is the same in-ear channel against the T7–T8 bipolar derivation.

Figure 4 highlights two complementary metrics for quantifying how well the in-ear EEG spectrum matches the corresponding scalp EEG electrode (T7/T8) frequency spectrum and the geometrically matched T7–T8 bipolar derivation. An average of 28.11% of 5 s time windows per subject were rejected for exceeding the 100 µV amplitude threshold. Figure 4 shows the signed Pearson correlation of log band power and Figure 5 the magnitude-squared coherence for each participant and condition, against the average-referenced temporal electrode and against the matched T7–T8 bipolar derivation. All 240 correlation values were positive except Participant 3 during eyes closed in the delta band. Across the full session, median correlation against T7 and T8 was 0.60 and 0.61 in delta, 0.45 and 0.46 in theta, 0.56 and 0.58 in alpha, and 0.38 and 0.42 in beta for the left and right ear respectively; against the matched T7–T8 derivation the corresponding medians were 0.66 and 0.62, 0.59 and 0.61, 0.74 and 0.62, and 0.47 and 0.50. Median full-session coherence against T7 and T8 was 0.46 and 0.48 in delta, 0.50 and 0.50 in theta, 0.46 and 0.49 in alpha, and 0.36 and 0.36 in beta; against T7–T8 it was 0.59 and 0.56, 0.67 and 0.58, 0.61 and 0.57, and 0.45 and 0.39. The matched bipolar derivation gave the higher value in 108 of 120 correlation cells and 118 of 120 coherence cells, and for the left ear in every band for every participant in the full-session coherence comparison.

### 3.5. Time-Series Comparison

**Figure 6:**
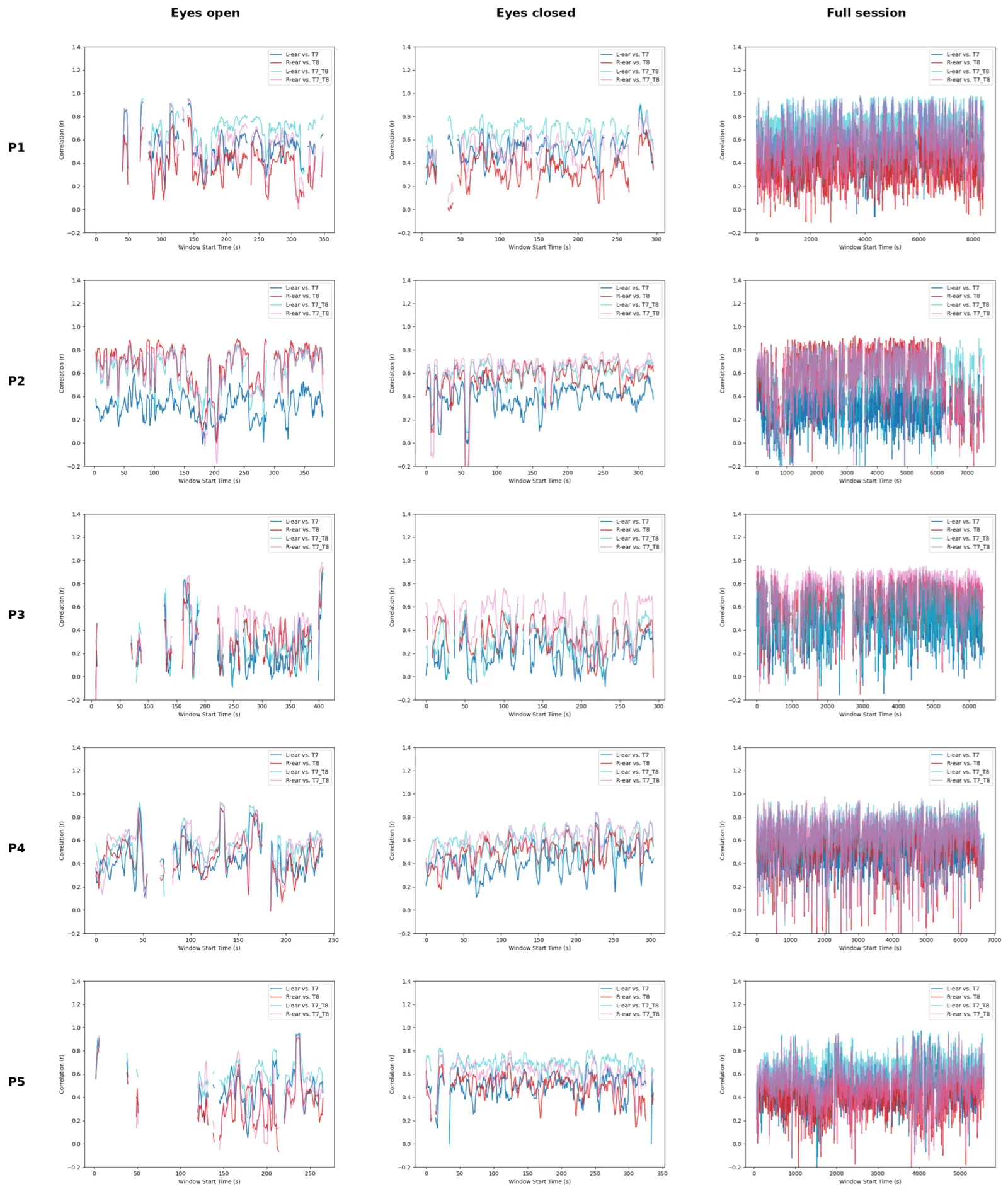
The sliding-window Pearson correlation between each in-ear channel and scalp EEG over time, for every participant (rows **P1** to **P5**) during eyes open (left column), eyes closed (middle column) and the full session (right column). Each panel shows the left ear against T7 (blue), the right ear against T8 (red), the left ear against the T7–T8 bipolar derivation (cyan) and the right ear against T7–T8 (pink), computed in 5 s windows with a 1 s step. The gaps are windows removed by the ±100 µV amplitude criterion. Note the different time axes: eyes-open and eyes-closed blocks are approximately five minutes, full sessions are 2.4 to 3.3 h.

To further investigate the signal fidelity, the temporal in-ear EEG signal was correlated to the temporal scalp EEG signal. An average of 4.11% of individual time points per subject were rejected for exceeding the 100 µV amplitude threshold. Within the ALL paradigm, across all 5 s windows, across all five subjects, the average left ear—T7 correlation was r = 0.43 ± 0.11 (ρ = 0.42 ± 0.11), while the average right ear—T8 correlation was slightly higher at r = 0.48 ± 0.08 (ρ = 0.48 ± 0.09). Figure 6 shows the sliding-window correlation over time for each participant and condition. In every participant and every condition, correlation with the matched T7–T8 bipolar derivation ran above correlation with the single average-referenced temporal electrode for most of the recording, reproducing in the time domain the pattern seen in band power (Figure 4) and coherence (Figure 5). Window-to-window variation was large: within a single multi-hour session, values ranged from near zero to above 0.9 in every participant, which is the within-session variability that the standard deviations above describe. Correlations were positive for the large majority of windows in all participants and conditions, with brief excursions below zero. Gaps in the traces are windows removed by the ±100 µV rejection criterion. Rejection was more frequent during the eyes-open blocks than the eyes-closed blocks, most markedly in Participants 3 and 5, consistent with ocular artifact during the eyes-open block. Participant 3 showed the lowest eyes-closed correlations, with the left ear against T7 rarely exceeding 0.4 in that block.

### 3.6. Comfort and Tolerability Assessment

**Figure 7:**
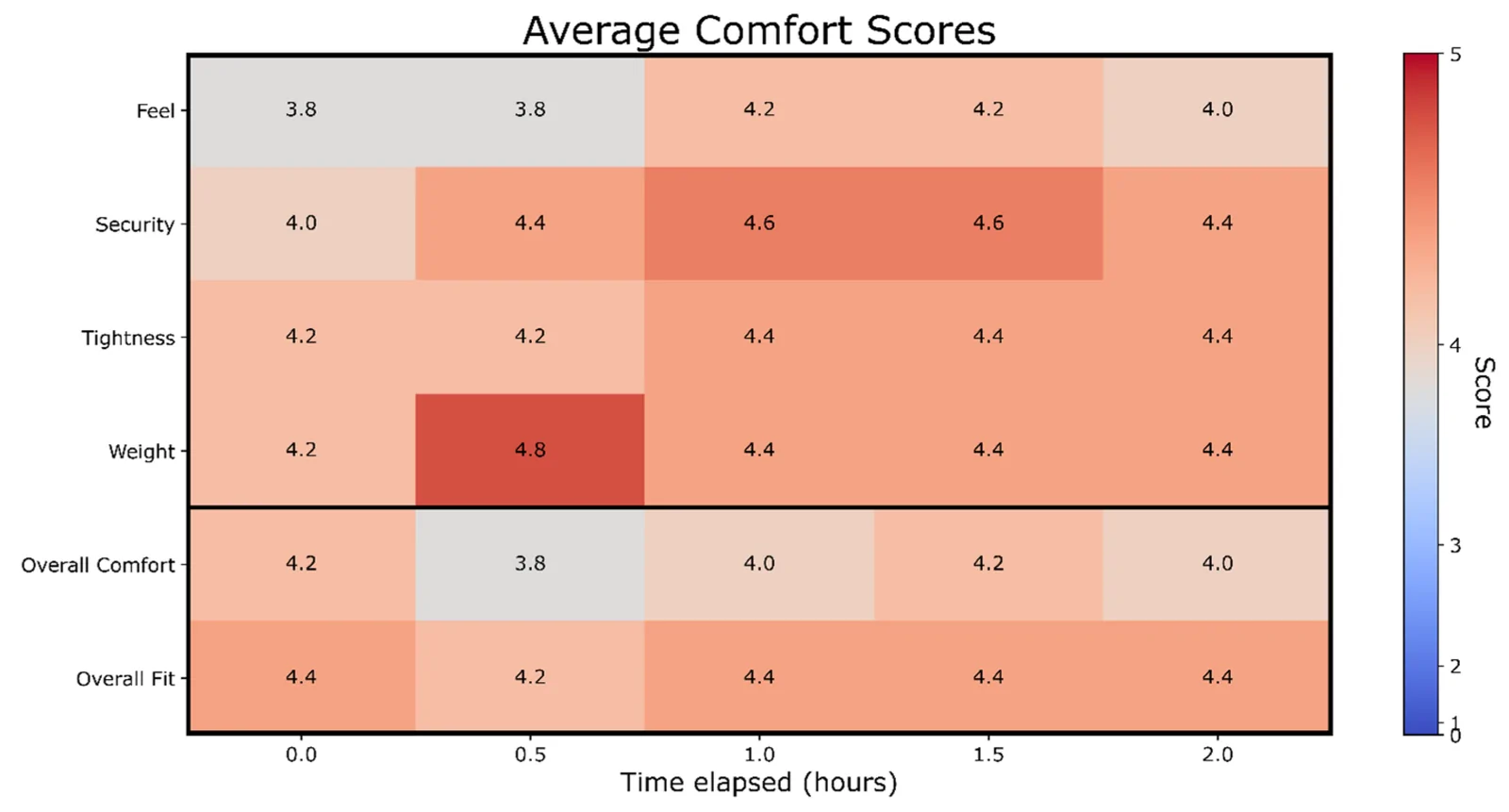
A time-series analysis of comfort-related parameters over the final two hours of each session, based on the average feedback from 5 participants; 0.0 corresponds to two hours before session end. Each parameter—feel, security, tightness, weight, overall comfort, and overall fit—is rated on a comfort scale, ranging from 0 to 5, where higher values indicate greater comfort.

Participant-reported comfort ratings over a 2 h wear period are presented in Figure 7. Device tightness remained stable at approximately 4.2 to 4.4 on a five-point Likert scale across all time points. The weight comfort scale increased from approximately 4.2 to 4.8 at the 30 min mark and subsequently returned closer to baseline by the end of the session. Security ratings increased from 4.0 to 4.6 during the first hour and then remained constant. Tactile feel improved steadily from an initial rating of 3.8 to 4.0+ over the course of two hours. Ratings for overall fit remained consistent between 4.2 and 4.4. Overall comfort began at 4.2, showed a slight decrease at the 30 min mark, and returned to 4.0 by the session’s end. Comfort ratings remained stable across the final two hours of each session, the window common to all five participants.

## 4. Discussion

All findings reported here are preliminary and exploratory. We hypothesized that in-ear EEG would show band-dependent correspondence with ipsilateral temporal scalp EEG, reproduce the direction of the eyes-closed alpha response, and be tolerated over multi-hour wear. With five participants that hypothesis is evaluated by description rather than by inference: where in-ear values are compared with scalp values, the individual participant values are shown and the number of participants showing the predicted direction is stated, so that the reader can judge consistency directly. Confirmation requires an adequately powered sample, which is the objective of our future study.

SeizEAR occupies a specific niche within the landscape of generic in-ear EEG systems. Prior generic designs, notably those from the Mandic and Muller groups, demonstrated that standardized earpieces can achieve adequate signal quality for research-grade applications including sleep staging, cognitive load monitoring, and drowsiness detection using minimal-contact or dry electrode configurations [23,24]. SeizEAR’s use of paste-based electrodes represents a deliberate trade-off within this design space: paste application adds a preparation step that limits fully autonomous deployment, but provides the low-impedance, stable contact needed for direct quantitative comparison against clinical-grade scalp EEG. This trade-off is appropriate for a supervised validation context where electrode preparation is already an accepted part of the recording workflow. Future iterations may explore hydrogel or semi-dry electrode alternatives that retain the impedance advantages of paste while reducing preparation time, building on the standardized sizing and cross-ear referencing architecture characterized in the present study.

By leveraging the anatomical advantages of the ear canal’s proximity to temporal brain regions while eliminating the need for custom fabrication, SeizEAR represents a step toward practical, wearable neurophysiological monitoring. This pilot study’s small sample size (n = 5) provides inadequate statistical power for definitive conclusions about device performance or clinical efficacy. The results should be interpreted as preliminary proof-of-concept findings requiring validation in larger, adequately powered studies. The device’s acquisition of in-ear EEG with moderate correspondence to scalp EEG, and its high comfort ratings, in a healthy volunteer sample in an off-the-shelf configuration addresses key barriers that have historically limited the clinical adoption of in-ear EEG technology. Our findings support the potential for the deployment of in-ear EEG that can enable continuous brain monitoring without the time-intensive setup, mobility restrictions, and social stigma associated with traditional scalp electrode arrays [3].

The development of the current SeizEAR design emerged through systematic evaluation of electrode configurations and contact methodologies. Initial attempts with dry electrodes, which were informal and not systematically characterized, proved inadequate for sustained signal quality; published dry-contact in-ear EEG implementations report impedance and signal-quality figures against which a paste-based design should be expected to improve in measurable terms [28]. We recognized that snug-fit positioning within the ear canal, combined with paste-based electrodes, creates a synergistic effect that enhances signal fidelity. Rather than treating canal conformance as a purely mechanical constraint, this design leverages the ear canal anatomy to establish stable contact. The conductive paste not only improves electrical conductivity but also compensates for minor anatomical variations within our standardized sizing system. This combination allows the device to maintain consistent electrode contact pressure and minimize movement artifacts while preserving the convenience of immediate deployment without custom molding procedures.

Band power in each canonical band was tracked across each session in 5 s windows. Window-to-window correspondence with scalp EEG varied widely within sessions (Figure 6), and time-resolved signal-quality metrics, dropout rates and early-versus-late comparisons were not computed, so stability of the signal across the session is not claimed. The correspondence observed is consistent with the previous literature establishing the validity of in-ear EEG for cortical activity measurement [15,29]. Establishing signal stability over multi-hour wear remains an objective for the follow-up study, as does evaluation for applications requiring long-term neurophysiological assessment, such as sleep monitoring or cognitive state evaluation [5,12,13,20].

Spectral power distribution analysis further supports that SeizEAR captures characteristic EEG frequency content. Specifically, delta band power was consistently higher in both left and right ear recordings compared to scalp averages and temporal electrodes (T7/T8), a finding we attribute to electrode–skin interface drift, low-frequency motion of the earpiece within the canal, and sweat-related potentials rather than to muscle activity: masticatory electromyographic activity concentrates well above the delta band, whereas the excess power here was confined to 1 to 4 Hz, consistent with the systematic characterization of physiological artifacts in ear versus scalp recordings by Kappel et al. [30]. Importantly, theta band power, critical for monitoring temporal lobe activity, was comparable between SeizEAR and T7/T8, consistent with the electrode placement; whether this reflects temporally localized sources cannot be determined from the present comparison, as discussed below [15]. Alpha and beta band powers were generally lower in the in-ear recordings than in traditional scalp EEG, which is consistent with known limitations of in-ear EEG in capturing posterior cortical rhythms and reflects the current literature [2,15]. Despite these differences, the pattern of spectral representation supports further evaluation of SeizEAR for monitoring clinically relevant brain activity.

When comparing the in-ear EEG time-series signal (L-ear/R-ear) with that of the corresponding scalp EEG signal (T7/T8), the mean correlation coefficient across windows was slightly above 0.4 on both sides of the head, indicating a moderate positive correlation. The similarity between Pearson and Spearman coefficients is consistent with a relationship that is approximately linear, not merely monotonic. The standard deviation across windows of approximately 0.1 describes variation within sessions rather than between participants; the per-participant traces in Figure 6 show the full range of that variation. The original submission inferred, from weak correlations with contralateral and averaged scalp channels, that each in-ear channel primarily tracks its local temporal electrode. That inference did not follow from the montage. The cross-ear channel is a wide bipolar derivation whose two electrodes sit on opposite sides of the head, and it is therefore sensitive to the lateral field gradient across the head rather than to a spatially restricted source. When the scalp signal is recomputed as a geometrically matched wide bipolar derivation, T7 referenced to T8, correspondence with the in-ear channels rises: the matched derivation gave the higher value in 108 of 120 band-power correlation cells and 118 of 120 coherence cells (Figure 4 and Figure 5), and ran above the single-electrode comparison for most of every recording in the time domain (Figure 6). The in-ear signal therefore corresponds more closely to a wide bipolar scalp derivation than to a single referenced electrode, and we describe it as such. The practical advantages of the cross-ear derivation are that it eliminates dependence on a scalp reference electrode and suppresses common-mode interference affecting both ears equally. Whether it additionally carries a preferential temporal contribution is a hypothesis for the follow-up study, in which correspondence with the full scalp array will be reported.

Subsequently, the frequency spectra of the in-ear EEG signal were compared to the corresponding scalp EEG signal. Pearson **r** correlation coefficients measure how well the window-to-window band powers of the two signals linearly track one another. Coherence (C) measures the strength of frequency-specific oscillatory coupling between the two signals, disregarding any stable phase offsets. The signal-to-noise ratio (SNR), a relative metric defined in Section 2, is derived directly from the Pearson coefficient, and is a key metric for quantifying how well the spectral activity of the in-ear EEG signal compares to the scalp EEG gold standard. The average SNR was higher in the delta and alpha bands, approximately −3 dB, and was lower in the theta and beta bands, approximately −6 dB. In the context of low-cost in-ear EEG electrodes, wherein <0 dB and even <−10 dB signal-to-noise ratios are common [9], this indicates moderate tracking of the corresponding scalp EEG by the in-ear EEG. Likewise, the coherence values were higher in the delta, theta, and alpha bands, and lower in the beta band. Coherence is reported without reference to a noise floor; the value of 0.25 given in the original submission was derived from an unverified assumption about conductive bridging within the earpiece and has been withdrawn. Coherence was consistently higher against the matched T7–T8 derivation than against the single temporal electrode in every band (Figure 5), indicating shared frequency-specific coupling that increases when the scalp comparator is geometrically matched to the in-ear derivation. Additionally, within the eyes-closed paradigm, the average alpha-band SNR increases substantially to ~0 dB, while the average alpha-band coherence did not substantially change. This discrepancy between SNR and coherence suggests the increase in alpha power during the eyes-closed paradigm within the in-ear EEG signal, providing further supplementary evidence that the in-ear EEG can respond to the alpha attenuation task.

The alpha attenuation ratios in Table 1 place this response in context. Posterior reactivity was the strongest in every participant who produced an alpha response, temporal reactivity was intermediate, and in-ear reactivity was the weakest, at roughly half the value of the matched T7–T8 derivation. This pattern has a straightforward explanation. Posterior alpha is largely symmetric and in phase across the two hemispheres, and a cross-hemispheric bipolar derivation suppresses activity that is symmetric across the midline; attenuated alpha reactivity is therefore what such a derivation should show, and the matched T7–T8 derivation shows the same attenuation relative to the single average-referenced electrode. The in-ear signal is not a global average of the scalp, which would reproduce posterior alpha strongly, but a lateral bipolar derivation, which by construction does not. Two participants merit individual comment. Participant 1 produced no eyes-closed alpha enhancement at any electrode, including O1 and O2, so the absence of an in-ear response in that participant reflects the absence of the physiological response itself rather than a property of the device. Participant 3 produced a normal scalp response with in-ear ratios below 1 in both ears, and the same participant’s eyes-closed block showed delta band correlations near zero or negative while alpha-band correlation was preserved and eyes-open and full-session values were unremarkable (Figure 4, Figure 5 and Figure 6). A loss of low-frequency correspondence and of alpha reactivity confined to the in-ear channels and to one five-minute block, with alpha correspondence preserved, is most consistent with low-frequency contamination of the in-ear signal during that block, for example from instability at the electrode–skin interface, rather than with a global failure of the earpiece or its fit.

Our results support cross-ear referencing as a workable signal processing paradigm for in-ear EEG applications [2,12,20]. This configuration reduces dependency on scalp reference electrodes, with signal patterns during resting-state and task-based recordings consistent with expected cortical activity. The potential implications extend beyond dedicated EEG devices to commercially available audio equipment, where dual-ear electrode integration could transform consumer headphones into neurophysiological monitoring platforms [14]. This approach can create opportunities for the integration of brain monitoring into everyday technology use while retaining a signal that corresponds moderately with scalp EEG [16].

T7 and T8 were initially selected as primary comparator channels on the basis of their anatomical proximity to the ear canal recording sites, consistent with prior in-ear EEG validation studies that use ipsilateral temporal electrodes as the nearest scalp reference [2,15]. De Fazio et al. provide a methodological counterpoint on impedance monitoring practice and on the behavior of in-ear derivations relative to frontal derivations under eyes-open, eyes-closed and evoked conditions [31]. Alpha modulation is strongest over the posterior cortex, and the present revision therefore reports alpha attenuation ratios at O1, O2, P7 and P8 alongside T7 and T8 (Table 2), and every correspondence measure against both the average-referenced temporal electrode and the geometrically matched T7–T8 derivation (Figure 4, Figure 5 and Figure 6). Correspondence with the full scalp array and with alternative references such as the ipsilateral mastoid is planned for the follow-up study.

The preliminary signal characteristics observed in this pilot study are consistent with the broader in-ear EEG literature, which has demonstrated utility across multiple applications including epilepsy monitoring [3,14,19]. The literature has established in-ear EEG utility for sleep disorder assessment, where unobtrusive recording enables naturalistic monitoring without the discomfort associated with traditional polysomnography [5,6,13,20,21,32]. Research has also shown in-ear EEG applications in cognitive load assessment and attention monitoring, with potential uses in workplace safety [12,33,34]. Emerging work demonstrates in-ear EEG capability for emotional state detection, relevant for mental health monitoring and therapy response assessment [4]. One of the most interesting developments for in-ear EEG research is in early dementia detection and cognitive decline tracking, where continuous monitoring could enable earlier intervention than currently possible with intermittent clinical assessments [14,16,35]. For epilepsy monitoring specifically, the literature supports in-ear EEG efficacy for seizure detection, particularly given the temporal lobe’s frequent involvement in epileptogenic activity [3,19]. The ability to provide continuous monitoring without mobility restrictions could improve seizure documentation and medication optimization for epilepsy patients.

Comfort assessment showed that participants consistently rated the device as well-tolerated across the final two hours of recording. All subjects reported that the earpieces felt sufficiently tight and secure for the duration of the session, addressing a requirement for practical clinical deployment. One particularly notable anecdotal observation was participants’ spontaneous comparison of SeizEAR to familiar audio earbuds, suggesting successful integration of medical functionality within a socially acceptable form factor. Comfort was evaluated at 30 min intervals; ratings were aligned to session end and reported over the final two hours of each session, the window common to all five participants, and conclusions about tolerability are limited to that window. This comfort was achieved using standardized small, medium, and large sizing options rather than custom-fitted devices, challenging the assumption that individualized molding is necessary for in-ear EEG applications [6,8]. This off-the-shelf approach mirrors successful commercial headphone design principles, thereby removing a barrier to widespread adoption.

Comfort trend analysis revealed several important insights. Ratings for tightness and overall fit remained stable throughout the 2 h sessions, indicating no perceived increase in discomfort or slippage over time. Security and tactile feel both improved with time, suggesting acclimatization and enhanced perception of stability. Overall comfort scores remained consistently high with only minor fluctuations, supporting further evaluation of the device for extended wear in clinical or real-world monitoring scenarios.

We observed instances of EKG artifact contamination in the EEG recordings, which initially presented challenges for signal interpretation. However, this observation reveals a potential feature of the in-ear EEG system. The co-occurrence of cardiac artifacts in the in-ear EEG recordings has been noted in the prior literature and, with appropriate signal separation techniques, may offer an opportunity for multimodal physiological monitoring in future device iterations [36]. The present study does not evaluate this capability. This capability would enable correlation analysis between neurophysiological and cardiac health parameters, expanding the device’s clinical utility beyond pure EEG monitoring. Such integrated physiological monitoring could provide valuable insights into the relationship between cardiovascular and neurological function, further enhancing the potential applications of ear-based bio-signal acquisition systems.

A notable trade-off inherent to the current SeizEAR design is the use of conductive paste for electrode–skin contact. While paste-based electrodes provide the low-impedance interface necessary for clinical-grade signal acquisition and direct comparison against scalp EEG, paste application adds a preparation step that is inconsistent with the long-term, unattended deployment that represents the broader promise of wearable in-ear EEG. Paste drying over extended recordings is a known limitation that can degrade signal quality and necessitates reapplication, constraining the practical wear duration in real-world settings. In the present study paste was not reapplied during any session, and because the acquisition platform reports impedance only as a threshold indicator, the time course of paste drying and the resulting operational window of the electrode could not be characterized. This trade-off was accepted in the present study, where the primary objective was signal fidelity investigation under supervised conditions rather than autonomous ambulatory deployment. Subsequent device iterations will evaluate semi-dry and hydrogel electrode alternatives that retain the contact quality of paste while reducing preparation burden and extending viable wear duration, building on the standardized sizing and cross-ear referencing architecture established here.

Several limitations must be acknowledged in interpreting our findings. The sample size of five healthy volunteers limits generalizability and statistical power for definitive conclusions about device performance. Several further limitations arise from the analysis. Numerical impedance values were not available from the acquisition platform, so the operational window of the paste-based electrode is not established. Blink removal by ICA was applied to the scalp signal only, which can lower but not raise the reported correspondence. The ±100 µV rejection criterion removes more windows from the higher-amplitude in-ear channels than from scalp, so surviving epochs are the quieter ones. Time-resolved signal-quality metrics, dropout rates and early-versus-late comparisons were not computed, and signal stability across the session is not claimed. Post-session otoscopic examination was not performed, so the endpoint reported here is tolerability rather than safety. Correspondence with the full 21-electrode array was not analyzed, and the claim that the in-ear signal preferentially reflects temporal cortex is therefore stated as a hypothesis rather than a finding. Downstream decoding was not benchmarked against scalp EEG; with five participants and no pathological data, a decoding benchmark would be a demonstration rather than a validation, and decoding performance is the primary outcome of the follow-up study. Additionally, our controlled laboratory environment did not include dynamic activities such as walking, running, or jumping that would stress-test the device’s capabilities during real-world use. The current prototype requires connection to external clinical recording equipment and does not yet represent a standalone ambulatory monitoring solution. Most importantly, this study focused exclusively on healthy participants and did not capture pathological brain states that are relevant to clinical application such as seizure detection. To address these limitations, we are developing a study which will evaluate SeizEAR performance in epilepsy patients within the epilepsy monitoring unit setting, with concurrent scalp EEG recordings serving as the clinical gold standard [3,19]. This future study will provide data on the device’s ability to detect seizure activity and other pathological patterns with protocol-defined otoscopic examination before and after each session as a formal safety endpoint in the target patient population. Future development may focus on miniaturization toward a fully integrated, wireless monitoring system capable of real-world deployment for continuous neurophysiological surveillance.

## Figures and Tables

**Table 1:** Participant characteristics: age at recording, sex, earpiece size (the same size was used in both ears), and completed recording duration. The protocol permitted sessions of up to six hours; all five sessions were concluded by the study team at between 2.4 and 3.3 h.

Participant	Age (y)	Sex	Earpiece Size	Completed Duration (h)
P1	24	F	S	2.4
P2	21	F	M	3.3
P3	21	M	M	3.2
P4	29	F	S	2.9
P5	21	M	M	2.9

**Table 2:** Eyes-closed to eyes-open alpha (8–13 Hz) power ratio per participant. Values above 1 indicate greater alpha power with eyes closed. In-ear channels use the cross-ear derivation. T7–T8 is a bipolar derivation geometrically matched to the cross-ear channel; all other scalp channels are common-average referenced. The median and range are across the five participants.

Channel	P1	P2	P3	P4	P5	Median	Range
**Left ear (cross-ear)**	0.81	1.58	0.50	1.15	1.23	1.15	0.50–1.58
**Right ear (cross-ear)**	1.21	1.77	0.70	1.49	1.74	1.49	0.70–1.77
T7	1.12	3.64	3.68	2.54	1.83	2.54	1.12–3.68
T8	0.78	3.61	2.93	2.19	2.37	2.37	0.78–3.61
T7–T8 bipolar	0.80	3.67	2.77	1.88	1.52	1.88	0.80–3.67
O1	0.92	6.51	9.99	7.67	5.68	6.51	0.92–9.99
O2	1.00	5.45	10.19	6.38	3.26	5.45	1.00–10.19
P7	0.81	9.26	6.61	4.99	3.94	4.99	0.81–9.26
P8	0.59	7.45	10.23	4.81	2.57	4.81	0.59–10.23
All scalp (mean)	0.86	4.61	6.23	3.55	2.96	3.55	0.86–6.23

## Data Availability

The datasets and analysis code used in this study are available from the corresponding author upon request.

## References

[1] Casson A.J., Yates D.C., Smith S.J.M., Duncan J.S., Rodriguez-Villegas E. (2010). Wearable Electroencephalography. IEEE Eng. Med. Biol. Mag..

[2] Mikkelsen K.B., Kappel S.L., Mandic D.P., Kidmose P. (2015). EEG Recorded from the Ear: Characterizing the Ear-EEG Method. Front. Neurosci..

[3] Zibrandtsen I.C., Kidmose P., Christensen C.B., Kjaer T.W. (2017). Ear-EEG detects ictal and interictal abnormalities in focal and generalized epilepsy—A comparison with scalp EEG monitoring. Clin. Neurophysiol..

[4] Athavipach C., Pan-ngum S., Israsena P. (2019). A Wearable In-Ear EEG Device for Emotion Monitoring. Sensors.

[5] Looney D., Goverdovsky V., Rosenzweig I., Morrell M.J., Mandic D.P. (2016). Wearable In-Ear Encephalography Sensor for Monitoring Sleep. Preliminary Observations from Nap Studies. Ann. Am. Thorac. Soc..

[6] Valentin O., Viallet G., Delnavaz A., Cretot-Richert G., Ducharme M., Monsarat-Chanon H., Voix J. (2021). Custom-Fitted In- and Around-the-Ear Sensors for Unobtrusive and On-the-Go EEG Acquisitions: Development and Validation. Sensors.

[7] Kondo S., Tanaka H., Dehais F. (2023). Improvement of the Accuracy of SSVEP-BCI with In-Ear EEG Using Multiple Regression Analysis. Neuroergonomics and Cognitive Engineering.

[8] Valentin O., Viallet G., Voix J. (2017). EARtrodes: Towards a wireless in-ear custom-fitted brain computer interface. Can. Acoust..

[9] Yarici M.C., Thornton M., Mandic D.P. (2023). Ear-EEG sensitivity modeling for neural sources and ocular artifacts. Front. Neurosci..

[10] Sintotskiy G., Hinrichs H. (2020). In-ear-EEG—A portable platform for home monitoring. J. Med. Eng. Technol..

[11] Bleichner M.G., Mirkovic B., Debener S. (2016). Identifying auditory attention with ear-EEG: cEEGrid versus high-density cap-EEG comparison. J. Neural Eng..

[12] Crétot-Richert G., De Vos M., Debener S., Bleichner M.G., Voix J. (2023). Assessing focus through ear-EEG: A comparative study between conventional cap EEG and mobile in- and around-the-ear EEG systems. Front. Neurosci..

[13] da Silva Souto C.F., Pätzold W., Wolf K.I., Paul M., Matthiesen I., Bleichner M.G., Debener S. (2021). Flex-Printed Ear-EEG Sensors for Adequate Sleep Staging at Home. Front. Digit. Health.

[14] Kaongoen N., Choi J., Woo Choi J., Kwon H., Hwang C., Hwang G., Kim B.H., Jo S. (2023). The future of wearable EEG: A review of ear-EEG technology and its applications. J. Neural Eng..

[15] Choi S.-I., Hwang H.-J. (2019). Effects of Different Re-referencing Methods on Spontaneously Generated Ear-EEG. Front. Neurosci..

[16] Mihai A.S., Geman O., Toderean R., Miron L., SharghiLavan S. (2025). The Next Frontier in Brain Monitoring: A Comprehensive Look at In-Ear EEG Electrodes and Their Applications. Sensors.

[17] Xu Y., De la Paz E., Paul A., Mahato K., Sempionatto J.R., Tostado N., Lee M., Hota G., Lin M., Uppal A. (2023). In-ear integrated sensor array for the continuous monitoring of brain activity and of lactate in sweat. Nat. Biomed. Eng..

[18] Meiser A., Bleichner M.G. (2022). Ear-EEG compares well to cap-EEG in recording auditory ERPs: A quantification of signal loss. J. Neural Eng..

[19] Joyner M., Hsu S.-H., Martin S., Dwyer J., Chen D.F., Sameni R., Waters S.H., Borodin K., Clifford G.D., Levey A.I. (2024). Using a standalone ear-EEG device for focal-onset seizure detection. Bioelectron. Med..

[20] Tabar Y.R., Mikkelsen K.B., Shenton N., Kappel S.L., Bertelsen A.R., Nikbakht R., Toft H.O., Henriksen C.H., Hemmsen M.C., Rank M.L. (2023). At-home sleep monitoring using generic ear-EEG. Front. Neurosci..

[21] Tang Y., Yin X., Wang X., Zhang Z. (2025). Application of Ear-EEG and Other Wearable Systems in Sleep Assessment. Appl. Comput. Eng..

[22] Kidmose P., Looney D., Jochumsen L., Mandic D.P. Ear-EEG from generic earpieces: A feasibility study. Proceedings of the 2013 35th Annual International Conference of the IEEE Engineering in Medicine and Biology Society (EMBC).

[23] Goverdovsky V., Looney D., Kidmose P., Mandic D.P. (2016). In-Ear EEG From Viscoelastic Generic Earpieces: Robust and Unobtrusive 24/7 Monitoring. IEEE Sens. J..

[24] Kaveh R., Doong J., Zhou A., Schwendeman C., Gopalan K., Burghardt F.L., Arias A.C., Maharbiz M.M., Muller R. (2020). Wireless User-Generic Ear EEG. IEEE Trans. Biomed. Circuits Syst..

[25] Gramfort A., Luessi M., Larson E., Engemann D.A., Strohmeier D., Brodbeck C., Goj R., Jas M., Brooks T., Parkkonen L. (2013). MEG and EEG data analysis with MNE-Python. Front. Neurosci..

[26] Delorme A., Sejnowski T., Makeig S. (2007). Enhanced detection of artifacts in EEG data using higher-order statistics and independent component analysis. Neuroimage.

[27] Lopez-Calderon J., Luck S.J. (2014). ERPLAB: An open-source toolbox for the analysis of event-related potentials. Front. Hum. Neurosci..

[28] Kappel S.L., Rank M.L., Toft H.O., Andersen M., Kidmose P. (2019). Dry-Contact Electrode Ear-EEG. IEEE Trans. Biomed. Eng..

[29] Moumane H., Pazuelo J., Nassar M., Juez J.Y., Valderrama M., Le Van Quyen M. (2024). Signal quality evaluation of an in-ear EEG device in comparison to a conventional cap system. Front. Neurosci..

[30] Kappel S.L., Looney D., Mandic D.P., Kidmose P. (2017). Physiological artifacts in scalp EEG and ear-EEG. BioMed. Eng. OnLine.

[31] De Fazio R., Yalçınkaya Ş.E., Cascella I., Del-Valle-Soto C., De Vittorio M., Visconti P. (2025). Forehead and In-Ear EEG Acquisition and Processing: Biomarker Analysis and Memory-Efficient Deep Learning Algorithm for Sleep Staging with Optimized Feature Dimensionality. Sensors.

[32] Mikkelsen K.B., Tabar Y.R., Kappel S.L., Christensen C.B., Toft H.O., Hemmsen M.C., Rank M.L., Otto M., Kidmose P. (2019). Accurate whole-night sleep monitoring with dry-contact ear-EEG. Sci. Rep..

[33] Kaveh R., Schwendeman C., Pu L., Arias A.C., Muller R. (2024). Wireless ear EEG to monitor drowsiness. Nat. Commun..

[34] Jeong D.-H., Jeong J. (2020). In-Ear EEG Based Attention State Classification Using Echo State Network. Brain Sci..

[35] Musaeus C.S., Waldemar G., Andersen B.B., Høgh P., Kidmose P., Hemmsen M.C., Rank M.L., Kjær T.W., Frederiksen K.S. (2022). Long-Term EEG Monitoring in Patients with Alzheimer’s Disease Using Ear-EEG: A Feasibility Study. J. Alzheimer’s Dis..

[36] Tamburro G., Stone D.B., Comani S. (2019). Automatic Removal of Cardiac Interference (ARCI): A New Approach for EEG Data. Front. Neurosci..

